# Damage in the Thalamocortical Tracts is Associated With Subsequent Thalamus Atrophy in Early Multiple Sclerosis

**DOI:** 10.3389/fneur.2020.575611

**Published:** 2020-11-17

**Authors:** Merlin M. Weeda, Ilanah J. Pruis, Aimee S. R. Westerveld, Iman Brouwer, Barbara Bellenberg, Frederik Barkhof, Hugo Vrenken, Carsten Lukas, Ruth Schneider, Petra J. W. Pouwels

**Affiliations:** ^1^Department of Radiology and Nuclear Medicine, MS Center Amsterdam, Amsterdam Neuroscience, Amsterdam UMC–Location VUmc, Amsterdam, Netherlands; ^2^Institute of Neuroradiology, St. Josef Hospital, Ruhr-University Bochum, Bochum, Germany; ^3^Institutes of Neurology and Healthcare Engineering, University College London, London, United Kingdom; ^4^Department of Neurology, St. Josef Hospital, Ruhr-University Bochum, Bochum, Germany

**Keywords:** multiple sclerosis, early MS, white matter damage, thalamus atrophy, thalamocortical tracts, fractional anisotropy, mean diffusivity, longitudinal study

## Abstract

**Background:** In early multiple sclerosis (MS), thalamus atrophy and decreased integrity of the thalamocortical white matter (WM) tracts have been observed.

**Objective:** To investigate the temporal association between thalamus volume and WM damage in the thalamocortical tract in subjects with early MS.

**Methods:** At two time points, 72 subjects with early MS underwent T1, FLAIR and diffusion tensor imaging. Thalamocortical tracts were identified with probabilistic tractography using left and right thalamus as seed regions. Regression analysis was performed to identify predictors of annual percentage change in both thalamus volumes and integrity of the connected tracts.

**Results:** Significant atrophy was seen in left and right thalamus (*p* < 0.001) over the follow-up period (13.7 ± 4.8 months), whereas fractional anisotropy (FA) and mean diffusivity (MD) changes of the left and right thalamus tracts were not significant, although large inter-subject variability was seen. Annual percentage change in left thalamus volume was significantly predicted by baseline FA of the left thalamus tracts *F*_(1.71)_ = 4.284, *p* = 0.042; while no such relation was found for the right thalamus. Annual percentage change in FA or MD of the thalamus tracts was not predicted by thalamus volume or any of the demographic parameters.

**Conclusion:** Over a short follow-up time, thalamus atrophy could be predicted by decreased integrity of the thalamic tracts, but changes in the integrity of the thalamic tracts could not be predicted by thalamus volume. This is the first study showing directionality in the association between thalamus atrophy and connected WM tract damage. These results need to be verified over longer follow-up periods.

## Introduction

Multiple sclerosis (MS) is an autoimmune disorder affecting the central nervous system. MS is characterized by atrophy in the gray matter (GM) and widespread pathology in the white matter (WM), which can be investigated with diffusion tensor imaging (DTI). From DTI, fractional anisotropy (FA) and mean diffusivity (MD) in WM can be estimated, which are both simplified measures for the overall integrity of WM (1, 2).

The thalamus, a central structure in the brain that acts as a convergence location as well as a gateway to the cortex (3), has numerous reciprocal WM connections to the cortex and subcortical structures, also referred to as the thalamocortical projections. These thalamocortical projections connect distinct thalamic nuclei to cortical areas, such as to the prefrontal and temporal cortex, to the posterior parietal cortex and to the somatosensory and motor/premotor cortices. (4). Due to its multitude of cortical connections the thalamus is involved in numerous neurological functions, such as motor, sensory, executive and higher cortical functions. Furthermore the thalamus plays a significant role in other functions, such as memory, emotion, attention and the regulation of sleep and wakefulness (3).

In subjects with MS, thalamic volumes are reduced when compared to healthy controls (5, 6) and have been found to decrease further, even early in the disease and over short follow-up periods (7–9). Moreover, thalamus atrophy is correlated with clinical disability (10, 11).

The thalamocortical projections exhibit higher lesion loads and more WM loss than non-thalamocortical projections in subjects with early MS (12, 13). In addition, subjects with early MS showed a correlation between reduced thalamic volume and reduced FA in the WM adjacent to the thalamus (14) or damage in the connected WM tracts, as seen by decreased FA and increased MD as well as increased WM lesion volume in the thalamocortical projections (13). Although some studies show a relationship between global WM damage and deep GM atrophy (15–17), no longitudinal studies have focused on damage of the WM specifically connected to these deep GM structures. Moreover, investigation of the directionality of the relationship between GM atrophy and WM damage remains scarce and inconclusive (13, 18–20).

Therefore, this study aims to investigate the longitudinal association between thalamus volume and WM damage in the connected tracts in subjects with early MS over a short follow-up. We hypothesize that damage in the thalamocortical WM is a predictor of thalamic atrophy, but that lower baseline thalamus volume does not predict increased thalamocortical WM damage over time.

## Methods

### Subjects

MS patients from a single center who were enrolled in a longitudinal MS cohort study (21, 22) and who underwent at least two MRI examinations including DTI were included for this study. Subjects included were all above 18 years of age and diagnosed with either RRMS based on the 2005 McDonald criteria within 2.5 years before the first MRI with DTI, or diagnosed with CIS within 1.3 years before the first MRI with DTI. Clinical performance was scored using the Expanded Disability Status Scale (EDSS) (23). Other parameters used for this study were: age, sex, disease duration (i.e., time from first clinical manifestation), treatment type (if applicable), and treatment duration (i.e., time on current treatment). The study was approved by the local institutional regulatory board (Reg.-No.:3714-10), and written informed consent was obtained from all individuals, according to the Declaration of Helsinki.

### MR Image Acquisition

Brain images were acquired on a 3 Tesla scanner (Philips Achieva, Best, The Netherlands) with a 32-channel head coil following a standardized imaging protocol with sequences covering the entire brain (24). For this analysis, we included the following acquisitions prior to contrast injection: (1) sagittal 3D T1-weighted fast field echo (FFE) (repetition time (TR)/echo time (TE)=8.5–10/4.6 ms; 180 slices; resolution =1.0 × 1.0 × 1.0 mm^3^; flip angle =8°); (2) sagittal 3D Fluid-attenuation Inversion Recovery (FLAIR) (TR/TE =4,800/286–323.5 ms; inversion time (TI) =1650 ms; resolution=1.0 × 1.0 × 1.0 mm^3^); and (3) transversal 2D echo-planar DTI (TR/TE = 7,000/90 ms; 2.5 mm slices; in-plane resolution =2.5 × 2.5 mm^2^, 32 volumes with b-value =900 s/mm^2^ and one volume, 2 averages, without diffusion weighting).

### MR Imaging Data Analysis

MR imaging data analysis was performed with FMRIB Software Library (FSL) version 5.08 and FreeSurfer version 6.0.

#### Structural Imaging

**Figure 1:**
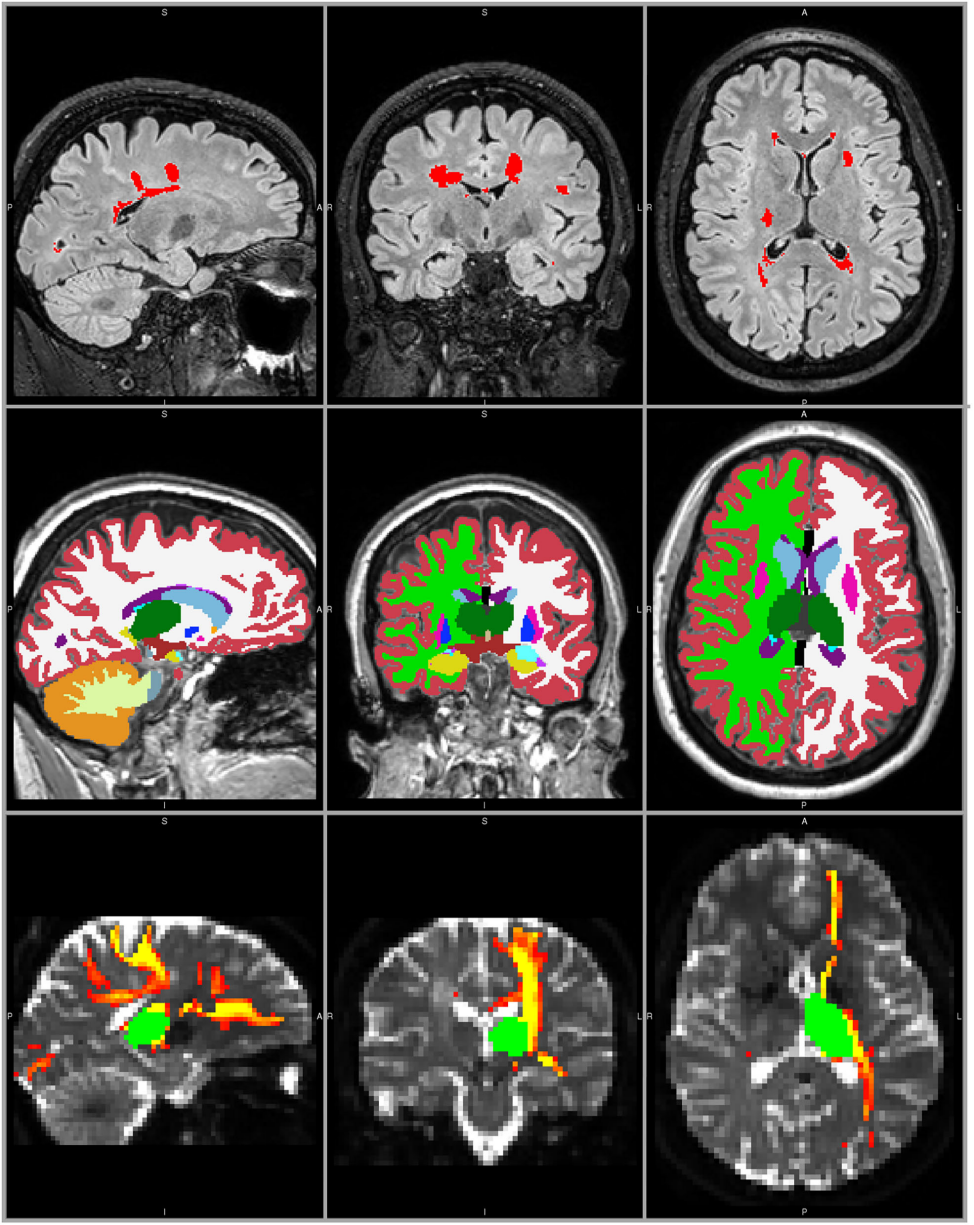
MRI processing example in a 28 year old female with RRMS. Top panels: T2-FLAIR weighted image with LST LPA lesion segmentation (red). Middle panels: T1-weighted lesion filled image with FreeSurfer segmentation, including left and right thalamus (dark green). Bottom panels: non-diffusion weighted image with the registered left thalamus (green) and the probabilistic connectivity distribution map of the corresponding thalamocortical tracts (red-yellow).

Brain extraction was performed on all T1w and FLAIR images using FSL BET (25), optimized for the current scan type and specifications (26). For lesion segmentation and subsequent filling on the T1w images with the signal intensity of the surrounding WM, Lesion Segmentation Toolbox with Lesion Prediction Algorithm (LST-LPA) was used (27, 28). Lesion-filled T1w images were processed with the longitudinal pipeline of FreeSurfer (29–31) and whole brain, WM, GM and left and right thalamus volumes were extracted. Volumes of whole brain, brain structures, and lesions were not normalized. An example of lesion segmentation on FLAIR images and FreeSurfer segmentation of brain volumes on T1w images is shown in Figure 1. Using FreeSurfer's transform matrices, segmentations were registered back to T1 native space with nearest neighbor interpolation, and subsequently linearly registered to DTI space with nearest neighbor interpolation.

All processed images were checked visually for potential errors in the segmentation of FreeSurfer and/or LST-LPA.

#### Diffusion Imaging

After correction for head movement and eddy current distortions (32) in the diffusion-weighted scans, the diffusion tensor was fitted with weighted least squares to obtain maps of FA and MD. The two-fiber model bedpostx (33) was used for estimation of the voxel-wise diffusion parameter distribution. Probabilistic tractography (probtrackx2) (34) was performed on the whole brain with the left and right thalami as separate seed regions. This probabilistic algorithm is capable of tracking fibers in the situation of multiple fiber orientations or crossing fibers. Its suitability has been specifically shown for tracking the thalamocortical projections (4). Streamlines crossing through the midline of the brain—apart from the corpus callosum, fornix, and brainstem—were rejected using a midline mask. Subsequently, a cerebral WM mask was applied to ensure no information from (sub)cortical or infratentorial structures was present in the thalamocortical tracts. An example of the resulting probabilistic connectivity distribution is depicted in Figure 1. This probabilistic connectivity distribution was used to obtain weighted mean FA and MD values per tract, in order to emphasize the major parts of the tract and decrease the effect of spurious streamlines. The entire tract (i.e., NAWM and lesions included) was used for further analysis.

### Statistics

Statistical analysis was performed in IBM SPSS Statistics for Windows, version 22.0 (IBM Corp., Armonk, N.Y., United States) and results were considered statistically significant upon *p* < 0.05.

To ensure that baseline differences would minimally influence the longitudinal measures, changes over time were calculated as a relative change with regard to baseline values. For equal comparison between subjects with different follow-up periods, these changes over time were converted to annual rates.

All parameters were tested for normality with a Kolmogorov-Smirnov test. Differences between baseline and follow-up were calculated using a paired samples *t*-test (nominal data) or a Wilcoxon signed ranks tests (non-nominal data). Differences between subjects with CIS and RRMS and between the left and right brain hemispheres were calculated using independent *t*-test (nominal data), Mann–Whitney *U*-test (non-nominal data) or Chi-square test (binomial data).

Forward linear regression was performed to see whether annual percentage change in thalamus volume could be predicted by baseline FA or MD of the thalamic tracts (left and right separately); and to see whether annual percentage change in FA or MD of the thalamic tracts could be predicted by baseline thalamus volume (left and right separately). All baseline demographics (age, sex, disease type and duration, treatment type and duration, EDSS) were included in the regression model as well, to correct for possible confounders.

## Results

### Demographics

From the total dataset of 82, ten subjects were excluded (received second line therapy *n* = 6; movement observed during MRI *n* = 3; erroneous lesion segmentation *n* = 1) resulting in a total of 72 subjects with early MS with a mean disease duration of 10.7 ± 8.9 months (range: 0.8 to 31.1 months) and a mean follow-up time of 13.7 ± 4.8 months (range: 5.1 to 27.3 months). The baseline demographics are shown in Table 1. Subjects with CIS had a significantly shorter disease and treatment duration than subjects with RRMS, but there were no differences between the disease types for any of the other demographics.

**Table 1:** Baseline demographics.

	**Total**	**CIS**	**RRMS**	***p*-value**
	***n* = 72**	***n* = 28**	***n* = 44**	
Age in years, mean ± SD	37.4 ± 10.9	37.5 ± 9.4	37.4 ± 12.0	*p* = 0.891^a^
Sex, m/f (%f)	28/44 (61%)	13/17 (57%)	15/27 (64%)	*p* = 0.513^b^
Disease duration in months mean ± SD (range)	10.7 ± 8.9 (0.8–31.1)	4.2 ± 3.7 (0.8–4.0)	15.2 ± 8.7 (1.1–31.1)	***p*** **< 0.001**^a^
Treatment, *n* (%)				*p* = 0.544^b^
None	17 (24%)	9 (30%)	8 (19%)	
Interferon	34 (47%)	15 (50%)	19 (45%)	
Glatiramer acetate	15 (21%)	4 (13%)	11 (26%)	
Dimethyl fumarate	5 (7%)	2 (7%)	3 (7%)	
Teriflunomide	1 (1%)	0 (0%)	1 (2%)	
Treatment duration in months mean ± SD (range)	4.1 ± 6.9 (0.0–27.3)	1.2 ± 3.1 (0.0–13.1)	6.2 ± 8.1 (0.0–27.3)	***p*** **= 0.011**^a^
EDSS, median with IQR	1.5 (1.5–2.5)	1.5 (1.5–2.5)	1.5 (1.0–2.5)	*p* = 0.494^a^
Follow-up time in months mean ± SD (range)	13.7 ± 4.8 (5.1–27.3)	12.5 ± 3.1 (7.9–25.4)	14.5 ± 5.7 (5.1–27.3)	*p* = 0.346^a^

SD, standard deviation; IQR, interquartile range.

Differences between CIS and RRMS tested by:

a *Mann–Whitney U-test*,

b *Chi Square test. p-values < 0.05 are highlighted in bold type*.

### Changes in Brain Volumes and WM Tract Integrity From Baseline to Follow-Up

Baseline and follow-up brain volumes and white matter measures are shown in Table 2. None of the measures differed between subjects with CIS or RRMS, therefore all analyses were performed in the entire cohort of subjects.

**Table 2:** Baseline and follow-up brain volumes and the FA and MD of the tracts connected to the thalamus, shown as mean with standard deviation, as well as the annual percentage change observed.

	**Baseline *n* = 72**	**Follow-up *n* = 72**	***p*-value paired samples *t*-test**	**Annual change (%, median with IQR)**
Whole brain volume	1140.3 ± 126.7	1131.5 ± 124.6	***p*** **< 0.001**	−0.65 (−1.52 – −0.01)
WM volume	446.5 ± 58.6	441.7 ± 58.1	***p*** **< 0.001**	−0.83 (−2.42 – +0.44)
GM volume	637.4 ± 68.6	631.9 ± 67.4	***p*** **= 0.002**	−0.93 (−2.03 – +0.44)
Thalamus volume				
Left	7.15 ± 0.91	7.06 ± 0.91	***p*** **< 0.001**	−1.34 (−2.14 – +0.43)
Right	6.88 ± 0.84	6.78 ± 0.83	***p*** **< 0.001**	−0.88 (−2.34 – +0.14)
Lesion volume	12.26 ± 10.10	11.73 ± 10.44	*p* = 0.053	−8.52 (−27.65 – +8.87)
FA				
Left	0.545 ± 0.027	0.546 ± 0.030	*p* = 0.624	+0.30 (−1.60 – +1.88)
Right	0.560 ± 0.030	0.558 ± 0.030	*p* = 0.493	−0.23 (−2.93 – +2.33)
MD				
Left	63.98 ± 3.63	64.39 ± 4.08	*p* = 0.354	+0.22 (−1.83 – +3.06)
Right	62.32 ± 3.90	62.91 ± 4.14	*p* = 0.148	+0.61 (−2.58 – +3.71)

*Volumes are in ml; MD is in 10^−5^ mm^2^/s. p-values < 0.05 are highlighted in bold type*.

Between baseline and follow-up, there were significant differences in whole brain volume (*p* < 0.001), WM volume (*p* < 0.001), GM volume (*p* = 0.002), and left and right thalamus volume (both *p* < 0.001). No significant differences over time were found for FA or MD of the thalamic tracts.

Due to varying follow-up times between the subjects, differences between baseline and follow-up were converted to annual percentage change, shown in the last column of Table 2, showing large inter-subject variation for the annual percentage change of thalamus volume and FA/MD of the connected tracts.

Furthermore, differences between the left and right hemisphere were observed for thalamus volume, FA and MD at baseline (all *p* < 0.001) and at follow-up (all *p* < 0.001). However, no significant differences between hemispheres were found for annual percentage change of thalamus volume, FA or MD.

### Relation Between Thalamus Volume and WM Integrity of Connected Tracts

**Figure 2:**
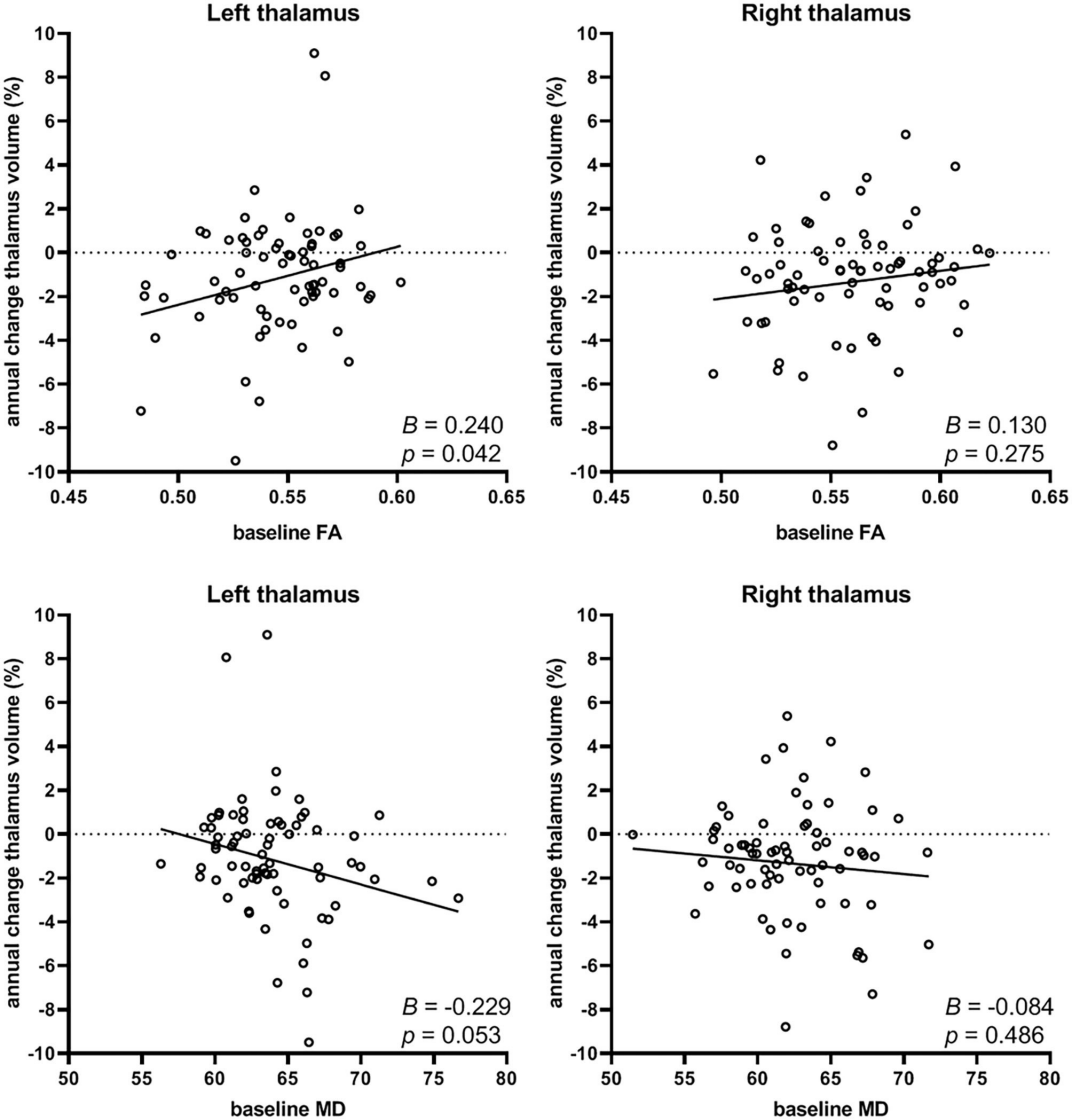
Relation between baseline FA (top row) or baseline MD (depicted as 10^−5^ mm^2^/s; bottom row) of the thalamocortical tracts and the annual percentage change in thalamus volume (left and right). Lower baseline FA (B=0.240, *p* = 0.042) was predictive of more negative annual percentage change of thalamus volume (i.e., atrophy), and a trend was seen for higher baseline MD (B=-0.229, *p* = 0.053, not significant) with increasing thalamus atrophy, but only in the left thalamus.

Linear regression analysis yielded a significant regression equation where annual percentage change in left thalamus volume was only predicted by baseline FA in the tracts connected to the left thalamus (*B* = 0.240, *p* = 0.042), and not by any of the demographic factors or by baseline MD, although a trend was seen for the latter (*B* = −0.229, *p* = 0.053). The resulting regression equation showed that lower baseline FA of the left thalamic tracts was predictive of more negative annual percentage change in left thalamus volume (i.e., atrophy): *F*_(1.71)_ = 4.284, *p* = 0.042 with adjusted *R*^2^ = 0.044. Scatterplots showing the relationship between baseline FA and MD with annual percentage change in thalamus volume is shown for left and right thalamus in Figure 2.

No significant regression equation could be computed for annual percentage change in right thalamus volume associated with baseline FA or MD, neither left nor right. Furthermore, we found no associations between the volumes of the thalami at baseline and annual percentage change in FA or MD. Thus, thalamus volume was not related to changes in white matter integrity over time.

## Discussion

This study aimed to investigate the longitudinal association between thalamus volume and white matter damage in the connected tracts. We hypothesized that damage in the thalamocortical WM is a predictor of thalamic atrophy, but that lower baseline thalamus volume does not predict increased thalamocortical WM damage over time.

Our results show that relative thalamus atrophy could be predicted only by baseline FA of the connected tracts, and not by any of the demographic parameters. This was found only for the left, and not the right, thalamus. Relative change in FA or MD of the thalamic tracts could not be predicted by baseline thalamus volume, or any of the other demographic parameters, neither left nor right.

Whole brain volume loss around 0.5% per year in our cohort was consistent with previous research [for meta-analysis, see (35)], as was the annual rate of thalamus atrophy of around 1.0% (9, 36) in early MS stages.

Previous cross-sectional studies showed a correlation between lower thalamus volumes and more damage in the white matter (13–15, 37–39), but no causality could be extracted from these studies. Regression analysis showed that lower white matter integrity (i.e., lower FA, and a trend for higher MD) could predict increased thalamus atrophy over time in our cohort. This suggests that damage of thalamocortical WM could have an effect on thalamic atrophy. Although this relation between baseline WM integrity and thalamic atrophy was not very strong, it points toward a disease mechanism of retrograde degeneration in subjects with MS, which has been proposed before (17, 20, 40–42). Beside the suggested phenomenon of Wallerian degeneration from distant WM lesions (43), our results in early MS patients with relatively low lesion volume, are in line with the indication of ongoing silent destructive processes of efferent and afferent thalamic projections which might induce measurable atrophy (14).

Since baseline thalamus volume and FA and MD of the connected tracts differed significantly between both hemispheres, this might explain why the relationship described above was only found for the left, and not right, thalamus. Leftward thalamic asymmetry could be demonstrated in healthy individuals and seems to corresponds to the physiological conditions (44). Interhemispheric asymmetry of brain diffusivity was described in normal individuals (45), as well as in MS, whereby increased apparent diffusion coefficients (ADCs) in the left thalamus were described (46), which is in line with our results demonstrating lower FA and higher MD values in the left thalamus. Longer follow-up periods are essential to further study the difference observed here between left and right thalamic tracts.

This study has some limitations. Since we had no healthy control group, we were not able to distinguish atrophy due to normal aging from pathological atrophy, although previous studies have shown that normal aging shows a lower atrophy rate (around 0.2 to 0.5% per year) than seen in our early MS cohort (47).

Second, we did not investigate normal appearing white matter and lesions separately, because this would lead to additional statistical tests and loss of power. However, we defined tract integrity by using FA and MD-values weighted by connectivity probability, such that the average weighted value is representative for the whole tract without lesion exclusion. Further, tensor elements were estimated with a slightly higher variance than ideal, since the DTI sequence contained one volume (2 averages) without diffusion weighting (*b* = 0 s/mm^2^), instead of an optimal number of 3 or 4 averages, considering the 32 volumes with diffusion weighting (48).

Last, this study was mainly explorative to find directionality and causality in the relationship between WM damage and GM atrophy. Longer follow-up is needed to confirm these results and to elucidate the differences between left and right thalamus. Future studies including larger sample sizes and extended follow-up could also be suited to elucidate the pathophysiological background in greater detail by investigating radial diffusivity and axial diffusivity as indicators for myelination and axonal damage.

In conclusion, this preliminary study in early MS with a short follow-up indicates that thalamus atrophy progresses faster upon more baseline damage in the connected thalamocortical white matter tracts. No significant relation was found for annual percentage change in FA or MD with thalamus volumes at baseline. However, the effect is small and therefore results should be confirmed over longer follow-up periods.

## Data Availability Statement

The raw data supporting the conclusions of this article will be made available by the authors, without undue reservation.

## Ethics Statement

The studies involving human participants were reviewed and approved by the local ethics committee of Ruhr-University Bochum (Approval No. 3714-10). The patients/participants provided their written informed consent to participate in this study and for the use and publication of their anonymized images and data.

## Author Contributions

MW, BB, HV, CL, and PP: conception of the work. MW and PP: design of the work. RS: patient recruitment and data acquisition. MW, IP, AW, IB, and PP: analysis of data. MW, IP, AW, BB, HV, RS, and PP: interpretation of data for the work. MW, BB, FB, HV, CL, RS, and PP: drafting and critical revision of the work for important intellectual content. MW, IP, AW, IB, BB, FB, HV, CL, RS, and PP: final approval of the version to be published. MW, RS, and PP: agreement to be accountable for all aspects of the work.

## Conflict of Interest

The authors declare that the research was conducted in the absence of any commercial or financial relationships that could be construed as a potential conflict of interest.

## Abbreviations

ADC: apparent diffusion coefficient
FA: fractional anisotropy
FFE: fast field echo
FMRIB: Oxford center for functional MRI of the brain
FSL: FMRIB software library
FLAIR: fluid-attenuated inversion recovery
GM: gray matter
IQR: inter quartile range
LST-LPA: lesion segmentation toolbox with lesion prediction algorithm
MD: mean diffusivity
NAWM: normal appearing white matter
TE: echo time
TI: inversion time
TR: repetition time
WM: white matter.
